# Supplementary material for: Impact of catch-up human papillomavirus vaccination on cervical conization rate in a real-life population in France
Source: PLoS One. 2022 Mar 11;17(3):e0264821. doi: 10.1371/journal.pone.0264821 (PMC8916646; doi:10.1371/journal.pone.0264821)
Supplement: Attachment — Submitted filename: Response to Reviewers.docx [file pone.0264821.s001.docx]

PONE-D-21-11439

Impact of catch-up human papillomavirus vaccination on cervical conization rate in a real-life population in France

PLOS ONE

Response to Editor and Reviewers

Answers are written in italics

Editor Comments:

3. Thank you for stating in the text of your manuscript "Data were extracted from the French National Health Insurance database called Echantillon Généraliste des Bénéficiaires (EGB), which is a permanent sample of the population covered by national health insurance". Please clarify whether this data was anonymized before researchers accessed it. Please also add all of this information to your ethics statement in the online submission form."

*EGB data is pseudonymized before researchers access it. There is one identifier per patient, but no identifying data.*

4. Thank you for including your manuscript text: "An institutional committee validates each research project." Please amend your current ethics statement to state:

a) whether ethics committee approval was specifically granted for this study

b) if so, include the full name of the ethics committee/institutional review board(s) that approved your specific study.

*The Institut Curie, as a cancer center, has access to EGB data. Access is however restricted to researchers from the Institute who have validated training provided by the Health Insurance. Each project with analysis of these data is reviewed by an internal committee of Ethics and Science in Institut Curie.*

5. We note that you have indicated that data from this study are available upon request. PLOS only allows data to be available upon request if there are legal or ethical restrictions on sharing data publicly. For information on unacceptable data access restrictions, please see http://journals.plos.org/plosone/s/data-availability#loc-unacceptable-data-access-restrictions.

In your revised cover letter, please address the following prompts:

a) If there are ethical or legal restrictions on sharing a de-identified data set, please explain them in detail (e.g., data contain potentially identifying or sensitive patient information) and who has imposed them (e.g., an ethics committee). Please also provide contact information for a data access committee, ethics committee, or other institutional body to which data requests may be sent.

b) If there are no restrictions, please upload the minimal anonymized data set necessary to replicate your study findings as either Supporting Information files or to a stable, public repository and provide us with the relevant URLs, DOIs, or accession numbers. Please see http://www.bmj.com/content/340/bmj.c181.long for guidelines on how to de-identify and prepare clinical data for publication. For a list of acceptable repositories, please see http://journals.plos.org/plosone/s/data-availability#loc-recommended-repositories.

We will update your Data Availability statement on your behalf to reflect the information you provide.

*This study involved third-party data (National Health Insurance – EGB). Therefore, we do not have the rights to share these third-party data, in accordance with regulations SNDS (Système National des Données de Santé).*

*Law governing the dissemination of SNDS data : L. 1461-1 III du code de la santé publique R. 1461-1 du code de la santé publique*

*For more information : https://www.snds.gouv.fr/SNDS/Finalites-autorisees*

*Therefore we are in the “a)” situation, with legal restictions on sharing a de-identified data set.*

*To access the EGB data, please visit this website to apply for dataset : https://documentation-snds.health-data-hub.fr/introduction/03-acces-snds.html#les-acces-permanents*

Additional Editor Comments (if provided):

P5L100 space issue “(24) .”

*Corrected*

P6 L110 decimal comma used in table 1

*Corrected*

P7 L141 Fig 2 flowchart is unclear. Right side box 1 requires women to be older than 25 in 2007. Next box states those are women born before 1991. However, a woman born in 1990 would be only 17 in 2007 instead of 25 as required by preceding box

*This is a typo, in the box to the right must be at least 25 years old in* ***2016*** *to be eligible for screening*

P7 L147 statistical test used for Table 2 (for example) is not listed. Please consider stating all p values without an exponent and list the test used at least once for each stretch of p values shown.

*All statistical tests were added in methods and in each table.*

*The R software cannot calculate a p-value lower than 2.2*10^-16^, as this value would not be very readable in a table we have replaced <2.2*10^-16^ by the value <10^-5^, which has a largely sufficient statistical value*

P8 L159. Fig 3 lacks legend at least for panels/parts of the figure

*Corrected*

P9 L171 Table 2 uses a thousand separator for large numbers, Tables 1 and 3 dont. Standard deviation is mentioned in the footer but not shown in Table 2.

*Corrected*

P9 L187 Fig 4 uses decimal points and decimal comma inconsistently

*Corrected*

P10L200 Table 3 it should probably be “mean age AT Pap/conization” instead of mean age of conization. In the header, non-vaccinated group is listed as “No-vaccinated”

*Corrected*

P11 L208 inconsistent use of brackets for references “[26–28].”

*Corrected*

P12 L246 it should probably be highlighted that most studies by other authors discussed in the discussion focused on 3 vaccine doses, while the current study mostly highlighted 1+ vaccine dose results

*A paragraph was added on this point to discuss the effect according to the number of doses administered P14 L263*

Reviewers' comments:

Reviewer #1:

page 3, line 39: It is stated that “Conization is part of the treatment of cervical dysplasia and cancer” … should probably be “Conization is part of the treatment of cervical dysplasia and early stage cancer

*The sentence has been corrected*

page 3, line 49: “group immunity” should rather be “herd protection” or “group pretection”

*Replaced by herd immunity*

page 3, line 55-57: Relevant to mention that recently published papers on real world effectiveness against cervical cancer from Sweden and Denmark.

*The reference has been added with a comment P3 L57*

page 7, line 131: it is stated “The main outcome was a reduction of the conization rate between the 132 non-vaccinated and vaccinated populations” should probably be

“The main outcome was a comparison of the conization rate between the 132 non-vaccinated and vaccinated populations”

*The sentence has been corrected*

Nice study, however, based on very few exposed cases (N=16). The limitations should also include the fact that it was not possible to adjust for differences between vaccinated and non-vaccinated.

In addition, it was not possible to consider age at vaccination, which has turned out to be an important factor when looking at effectiveness.

*The manuscript has been revised to discuss these two points P14 L273 and 259*

Have the authors tried to restrict the analysis even further to birth cohorts with higher coverage – e.g. birth cohorts 1989-1991? or will you then loose too much power.

*The exclusion of cohorts poses several problems: decrease of power, the study is no longer addressing all catch-up vaccination but just a part, increase of family-wise error rate.*

The women belonging to birth cohorts with a low coverage may have specific reasons to be vaccinated – in other words, they may have a different behavior/risk factor profile linked to a higher or lower exposure to HPV pre-vaccination

*The increase of the vaccine coverage in time is explained by the fact that there is no vaccine campaign but a vaccine by individual approach (coming from the doctor or the patient), therefore the implementation of the vaccination is slow. This low vaccine coverage is addressed in the introduction, we have developed this "French" problem in the discussion P15 L302 with references added*

Reviewer #2:

The authors state on p5 that Gardasil AND Cervarix were the vaccines offered over the period of the evaluation, but I could not see any information as to what the estimated relevant proportions of the separate vaccines were. This would be valuable data as there is some evidence to indicate differential performance of the vaccines particularly in relation to cervical disease outcomes

*Unfortunately, the only information available in the data extracted from this reimbursement database was the number of dose and the dates of dispensing. The type of vaccine was not included in the data.*

The authors do not present information on deprivation - was a proxy of this not available from the national data set?

*The EGB is a health database that does not contain socio-economic data*

I understand that it is not a key deliverable of the manuscript but some more discussion of why vaccine uptake rates are low in France compared to other countries would be welcome and also implications for herd immunity (or lack thereof)

*There is no vaccine campaign in France but a vaccine by individual approach (coming from the doctor or the patient). This low vaccine coverage is addressed in the introduction, we have developed this "French" problem in the discussion P15 L302*

There are a couple of unusual phrases - eg page 12, line 250 "HPV contamination" arguably should be "HPV exposure" - I am also not sure what a "General scheme population" is, as described on page 13

*The word has been replaced. The notion of general regime was clarified in the discussion: it corresponds to more than 92% of the population, i.e. less than 8% excluded*
