# Supplementary material for: Impact of catch-up human papillomavirus vaccination on cervical conization rate in a real-life population in France
Source: PLoS One. 2022 Mar 11;17(3):e0264821. doi: 10.1371/journal.pone.0264821 (PMC8916646; doi:10.1371/journal.pone.0264821)
Supplement: Attachment — Submitted filename: Response to Reviewers.docx [file pone.0264821.s002.docx]

PONE-D-21-11439

Impact of catch-up human papillomavirus vaccination on cervical conization rate in a real-life population in France

PLOS ONE

Response to Reviewers

Answers are written in italics

4. Have the authors made all data underlying the findings in their manuscript fully available?

The PLOS Data policy requires authors to make all data underlying the findings described in their manuscript fully available without restriction, with rare exception (please refer to the Data Availability Statement in the manuscript PDF file). The data should be provided as part of the manuscript or its supporting information, or deposited to a public repository. For example, in addition to summary statistics, the data points behind means, medians and variance measures should be available. If there are restrictions on publicly sharing data—e.g. participant privacy or use of data from a third party—those must be specified.

Reviewer #1: Yes

Reviewer #2: No

*The reviewer did not specify in his comments why he disagrees on this point. All necessary information has been provided in the texts and forms regarding restrictions on publicly sharing data*

________________________________________

6. Review Comments to the Author

Please use the space provided to explain your answers to the questions above. You may also include additional comments for the author, including concerns about dual publication, research ethics, or publication ethics. (Please upload your review as an attachment if it exceeds 20,000 characters)

Reviewer #1: The authors have adequately responded to the comments. However, the new sentence on p. 3:

"More recently, a large Swedish cohort study showed a reduced risk of invasive

cervical cancer among girls and women vaccinated (20)" should be something like ...

So far, only two large cohort studies studies - one from Swedish (20) and one from Denmark (ref) have shown a reduced risk of invasive cervical cancer among girls and women vaccinated"

Ref: Real-World Effectiveness of Human Papillomavirus Vaccination Against Cervical Cancer.

Kjaer SK, Dehlendorff C, Belmonte F, Baandrup L.

J Natl Cancer Inst. 2021 Oct 1;113(10):1329-1335

As cervical cancer is the ultimate end point, it is important to mention both of the only two papers which have currently documented effectiveness in this regard.

*The Danish study has been added to the manuscript, thank you for this comment.*

Reviewer #2: The manuscript is improved as a result of the revision(s) - i also accept the limitations the authors explain in relation to dissemination of the original data

*Thank you.*
